# Extract from a Letter by the Senior Editor

**Published:** 1859-09

**Authors:** 


The following is an extract from a letter by the Senior Editor, dated Boston, the 14th of August:—

Yesterday I spent two hours at the Massachusetts General Hospital, Dr. J. Mason Warren having kindly called for me. While there I was introduced to Professor Henry J. Bigelow, Dr. Townsend, Dr. Cabot, Dr. Gay, and Dr. Clark, all officers of the institution. Dr. Gay extirpated a cancerous breast, in a state of ulceration, from a woman about forty-five years old, otherwise apparently in good health. He was followed by Dr. Clark, who amputated the thigh of a youth of twenty, on account of encephaloid disease of the lower part of the femur, first noticed last February. The operation was performed by the flap-method, by cutting from without inward. Next, Dr. Clark extirpated a testicle from a young man who had formerly had gonorrhœa. The organ began to enlarge a year ago, and was about the size of a goose egg. The upper part of the tumor fluctuated very distinctly, while the lower was very hard. The whole swelling had a uniform smooth appearance; the spermatic cord was sound, and there was no enlargement of the subcutaneous veins. A free incision being made, commencing near the external ring, a large quantity of water flowed out, leaving the testicle, hard and somewhat enlarged, exposed below. Upon consultation with one of his colleagues, Dr. Clark removed the organ, applying a stout ligature to the spermatic cord.

The next case was one of urinary calculus, a young man from the interior of Massachusetts, upon whom Professor Bigelow had already performed lithotropsy twice, the first operation having been executed just a week ago, and the second three days after that. The Heurteloup crusher having been introduced, the instrument was carried about in every direction, but failed to detect any fragments of stone.

All these patients were under the influence of ether, and the operations were performed with great neatness, although not very rapidly. The stone in Dr. Bigelow's case was one of triple phosphate, and the fact that he succeeded in getting rid of it at two sittings shows him to be an operator of no ordinary skill.

The amphitheatre of the hospital, planned by the late Professor Warren, is a very neat and pretty room, capable of seating about two hundred students, remarkably well lighted, and provided with a spacious area. Arranged along the wall of the room is a large open case filled with instruments, some of them deposited by the first Warren, and others by his son, who was so long Professor of Anatomy and Surgery in Harvard University, and one of the original surgeons of the hospital. Among other curiosities is a saw, invented by Dupuytren, for dividing the bone in amputation, and, at the same time, holding back the flaps. It was intended to obviate the necessity of assistants on the field of battle.

One of the most curious objects in the amphitheatre is an operating table, devised by Professor Henry J. Bigelow. It is altogether a remarkable piece of mechanism, so arranged by means of screws and hinges as to permit the patient, in a few seconds, to be placed in any position that may be most desirable. The original cost was $250, but I was told that it is now manufactured at about one-half that price. It certainly possesses great advantages, and no operating room should be without it.

The Massachusetts General Hospital contains about one hundred and forty beds, and is one of the neatest institutions of the kind in the world. It is well ventilated, and nothing could be more clean and orderly. The medical board consists of twelve members, six surgeons and six physicians, two of each of whom are on duty at the same time for a period of four months, the patients being assigned alternately as they are admitted. This arrangement greatly lightens the labors of the professional attendants, at the same time that it contributes very much to the welfare of the inmates.

Among the more interesting cases in the surgical ward were two of resection of the knee-joint; one of ununited fracture of the humerus, of twelve months standing, cured by Dr. Warren by the seton; and one of cancer of the lower lip, operated upon nearly a fortnight ago by Dr. Townsend, the lost substance having been replaced by Erichsen's method. One of the cases of resection of the knee, a young girl of about eighteen, was gradually sinking under purulent infiltration of the thigh, the swelling of which was enormous. This was the more surprising, as the operation had been performed nearly three months ago. The other case, that of a girl of thirteen, also operated upon twelve weeks ago, was doing well, with the prospect of a useful limb. Excision of the knee has now been performed five or six times at the hospital, with, in the main, a very gratifying result.

Close by the Massachusetts General Hospital stands the Medical Department of Harvard University, a small, unpretending edifice with admirable internal arrangements. The amphitheatre, the only lecture-room that I saw, is a beautiful little room, well lighted, and excellently adapted to the objects for which it is intended. Dr. Bigelow showed me the beautiful drawings with which he illustrates his courses of lectures in the college. They are of colossal size, splendidly colored, and the most graphic specimens of art of the kind that I have ever seen. They were made by a native American, under Dr. Bigelow's immediate superintendence, at a cost of from between $3000 and $4000.

Here, working, coat off, in a room by himself, I found my old friend, Dr. J. B. S. Jackson, the Professor of Pathological Anatomy, distinguished not less for his modesty and great kindness of heart than for the extent and variety of his attainments; the very pattern of an upright, useful, and industrious man, one of whom any school and any city might justly be proud. The lapse of twenty years, when I first saw him, has wrought a silvery change in his hair, but in all other respects he is the better man for his increased years. Having but little practice, he devotes himself almost exclusively to pathological pursuits, and there is no man, not even Rokitansky himself, who exhibits a greater interest in a morbid specimen.

The younger Warren—if a man who has just become a grandfather may be so called—worthily represents the honor of his illustrious descent, being the third of his family who have shed lustre upon our profession, resembling in this respect the Meckels and the Monros. As already stated, Dr. J. Mason Warren, although not a professor, is connected with the Massachusetts General Hospital, and in the enjoyment of a large private surgical practice. The heir of a great name, he seems to be full of enthusiasm, and to spare no pains to extend his professional reputation. While at his house a few mornings since, he showed me the specimen of a case, which, forty years ago, attracted a vast deal of attention, from the respectability of the patient, the obscurity of the diagnosis, and the reputation of some of the medical witnesses, especially of the elder Warren, who made the case afterwards the subject of an elaborate paper addressed to the presiding judge, Chief Justice Parker. Dr. Warren diagnosticated the injury as a dislocation into the lower and outer part of the thyroid foramen, with a portion of the femur resting upon the tuberosity of the ischium; and the dissection made three months ago shows that his opinion was well founded, only that the bone lay more immediately in the thyroid foramen than he had been led to suppose, but into which, as his son conjectures, it might have been easily forced in the subsequent attempts at reduction. The attending surgeon, who had mistaken the case for one of fracture of the neck of the thigh-bone, was sued for malpractice, but was ultimately acquitted on account of the diversity of the views of the medical witnesses. The specimen, which was preserved at great trouble and expense, exhibits a new osseous socket, forming a complete wall for the head of the femur, nearly three inches in depth, and provided with several adventitious ligaments. The acetabulum is nearly entirely obliterated. The new joint admitted of useful motion.

Dr. Warren also showed me the skull and heart of the immortal Spurzheim, who died at Boston in 1832, and whose other remains lie at Mount Auburn, beneath a marble monument modeled after that of Scipio Africanus. It bears simply the name of the illustrious founder of phrenology. The skull of Spurzheim is well formed, but is not particularly large. The organ of veneration must have existed in a high degree, and amativeness was also quite large. The heart was of extraordinary size.

I could not, of course, pass through Boston without paying my respects to that good man and excellent physician, Dr. James Jackson, now upwards of eighty years of age, but still erect and vigorous, and full of animation. He received me with much cordiality, and, during the half hour which I spent with him, he alluded with great kindness to some of my colleagues, especially Professors Meigs and Dunglison, with both of whom he is personally acquainted. Notwithstanding his advanced age, this venerable physician is still able to attend to practice, his intellect and judgment being unimpaired and unclouded. Long may they remain so!

Dr. Hayward has just returned, after an absence of two years, from Europe, where he received much attention from his professional brethren. He looks healthy and vigorous, and bids fair to reach a ripe old age. There are few men in the United States who have reflected greater credit upon American surgery than he. Although he has not published much, yet whatever he has written has been eminently judicious and useful. He retired from practice a few years ago, in the enjoyment of honor and wealth.

